# Combined Analysis of Primary Metabolites and Phenolic Compounds to Authenticate Commercial Monovarietal Peach Purees and Pear Juices

**DOI:** 10.3390/molecules24183289

**Published:** 2019-09-10

**Authors:** Antoni Delpino-Rius, Jordi Eras, Ferran Gatius, Mercè Balcells, Ramon Canela-Garayoa

**Affiliations:** 1 Department of Chemistry, University of Lleida-DBA, R+D Tecnio Center, Avda. Alcalde Rovira Roure, 191, E-25198 Lleida, Spain (A.D.-R.) (F.G.) (M.B.) (R.C.-G.); 2 Scientific Technical Service DATCEM, University of Lleida, Avda. Alcalde Rovira Roure, 191, E-25198 Lleida, Spain

**Keywords:** authentication, PCA, primary metabolites, phenolic compounds, NMR, UPLC-PDA-MS/MS

## Abstract

Here we authenticated single-varietal peach purees and pear juices on the basis of primary metabolite and phenolic compound analysis by Proton Nuclear Magnetic Resonance (^1^H-NMR) and Ultra Performance Liquid Chromatography coupled to Photodiode Array and Tandem Mass Spectrometry (UPLC-PDA-MS/MS), respectively. After suitable preprocessing, the ^1^H-NMR and chromatographic data were evaluated by principal component analysis (PCA). The PCA combining data from primary metabolites and phenolic compounds allowed the separation of the clusters in all cases, allowing discrimination of processed and unprocessed peach purees, both separately and pooled. The PCA of primary metabolites allowed the cluster separation of purees of distinct peach varieties but not between processed and non-processed purees. The PCA of phenolic compounds allowed better cluster separation than of primary metabolites. For pear juices, both PCA approaches allowed satisfactory discrimination of Alejandrina, Conference, and Blanquilla cultivars. These approaches may help to better control cultivar authenticity in fruit products. It could therefore contribute to the development of a process to achieve products characterized by a quality characteristic of a given cultivar.

## 1. Introduction

Peach and pear are two of the most widespread fruit crops in Southern Europe. Consequently, the production of processed products (fruit juice, jam, nectar…) is an important industrial activity in this region. Peach flavor (3.7%) ranks fourth in the juices and nectars most consumed in the European Union (EU), behind orange (38.5%), mixtures of flavors (19.9%), and apple (13.3%). Pear is included in the 21% of other flavors consumed, although it is noteworthy that in countries such as Italy, pear accounts for market shares of up to 12.5% [1]. To attract consumers, producers tend to be highly innovative when offering new products. Indeed, they have recently developed single-varietal juices. Juices are valuable commodities and economic fraud in the fruit juice sector is widely reported [2,3], for example adding a co-fruit, a less expensive fruit, or the juice of a fruit that is easier to find [4,5]. In this context, the commercialization of monovarietal juices opens up further opportunities for fraud, while increasing difficulty in detection. Therefore, novel methods to control their authenticity are welcome and interesting for consumers. In this regard, knowledge of the chemical composition of each cultivar and the effect of blending on this composition can contribute to the production of processed products with controlled quality.

To authenticate and characterize fruit cultivars, several techniques have been applied, such as liquid chromatography [6], gas chromatography [7], near infrared spectroscopy [8], nuclear magnetic resonance [4,9,10], and mass spectrometry [10]. These techniques allow the analysis of various metabolites, such as amino acids, organic acids, sugars, phenolic compounds, and carotenoids. Nuclear Magnetic Resonance (NMR) and Liquid Chromatography coupled to Photodiode Array and Tandem Mass Spectrometry (LC-PDA-MS) are especially suitable for the analysis of food and drink, since both have excellent reproducibility and generally have minimum sample preparation requirements. NMR spectroscopy has been used to analyze primary metabolites (amino acids, organic acids, and sugars), while LC-PDA-MS has been successfully applied for the analysis of phenolic compounds in fruit samples.

Several studies have addressed the characterization and classification of peach cultivars on the basis of their phenolic profile [11,12,13]. NMR has also been applied to compare two varieties of peach with distinct resistance to pests [14]. Moreover, pear cultivars have been described on the basis of their phenolic profile, mainly their flavonol glycoside composition [15,16,17]. For peach and pear juices, the effects of the different processing steps on the final compounds have also been described [18,19,20,21].

All previous studies were aimed at differentiating fruit cultivars using juices prepared in the laboratory. We wanted to evaluate whether industrially obtained juices and purees maintain enough characteristic compounds to differentiate between cultivars using common analytical tools. Consequently, here we seek to compare and combine NMR for primary metabolites and Ultra Performance Liquid Chromatography coupled to Photodiode Array and Tandem Mass Spectrometry UPLC-PDA-MS for phenolic compounds to determine whether these analytes allow differentiation of cultivars used for the preparation of peach puree and pear juice. This approach could improve the control of cultivar used in the preparation of targeted fruit products. Hence, the method could contribute to the development of processes devoted to the preparation of products with a sensory quality characteristic of a given cultivar.

## 2. Results and Discussion

### 2.1. ^1^H NMR and Chemometric Analysis of Primary Metabolites

^1^H NMR analysis of the peach puree and pear juices allowed the identification of primary metabolites. Sugars, organic acids, and amino acids were the metabolites identified in fruits. A pH of 6.5 was selected on the basis that it gave the best resolution among the signals of the main compounds along the ^1^H spectrum. The signals of these compounds were identified in the ^1^H spectrum by their diagnostic signals [14] and confirmed by spiking samples with standards. The characteristic signals of alanine (ALA)(β-CH_3_) and asparagine (ASP)(β, β′-CH_2_) were also identified in the ^1^H spectrum as free amino acids. Table 1 shows the chemical shifts of the signals of the metabolites selected. Maleic acid (2 x α-CH) at 6.2 ppm was used as internal standard. To assess the suitability of this acid as an internal standard for the compounds chosen, a pear juice sample was fortified at various concentrations with the sugars, organic acids, and amino acids identified. The behavior of all the compounds was linear over the range, thereby confirming that maleic acid at a concentration of 15 g/L is a suitable internal standard for fruit samples. The ratio between the metabolite signal area and signal area of maleic acid at 6.2 ppm was used to build the PCA model.

The PCA was applied to determine the main sources of variability present in data sets and to establish the relationship between samples (objects) and primary metabolites (variables). The weight of variables was normalized by the ratio of their standard deviation in the data set. In both models, the first two PCs (PC1 and PC2) plotted were selected to provide the highest variation of data objects (PC1 and PC2 explained 43% and 33% of the information for peach purees and 60% and 24% for pear juices, respectively).

**Figure 1:**
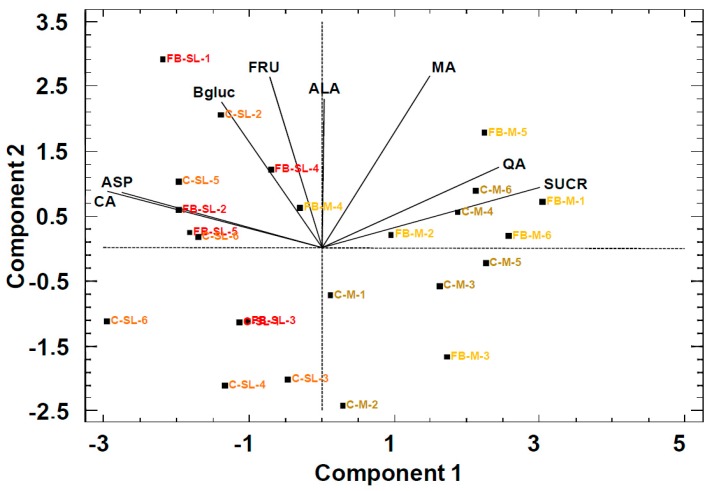
**Principal Component Analysis** (PCA) bi-plot of peach purees with primary metabolites as statistical variables. FB: Freshly blended, C: Commercial, SL: Spring Lady cultivar, and M: Miraflores cultivar. Ending number is the batch number. PC1: 43%, PC2: 33%.

The scores of the data presented in the PCA bi-plot (Figure 1) for peach purees showed a moderate separation through PC1 between the Miraflores (M) and Spring Lady (SL) cultivars in both commercial (C) and freshly blended (FB) purees. 

The loadings of the variables used in the model reveal that the separation was on the basis of citric acid (CA) and asparagine (ASP) against sucrose (SUCR), quinic acid (QA) and malic acid (MA) (Pearson correlation –0.68, *p* < 0.05). The concentrations in Spring Lady (SL) of citric acid (CA)) and asparagine (ASP) were higher than in Miraflores (M). In contrast, sucrose (SUCR) and quinic acid (QA) concentrations were higher in Miraflores (M). PCA scores did not show a separation between commercial (C) and freshly blended (FB) peach purees in spite of the cultivar. The information in the PCA model was consistent with previous results, which showed no effect of fruit processing on the primary metabolites detected by ^1^H NMR in the juices [22].

**Figure 2:**
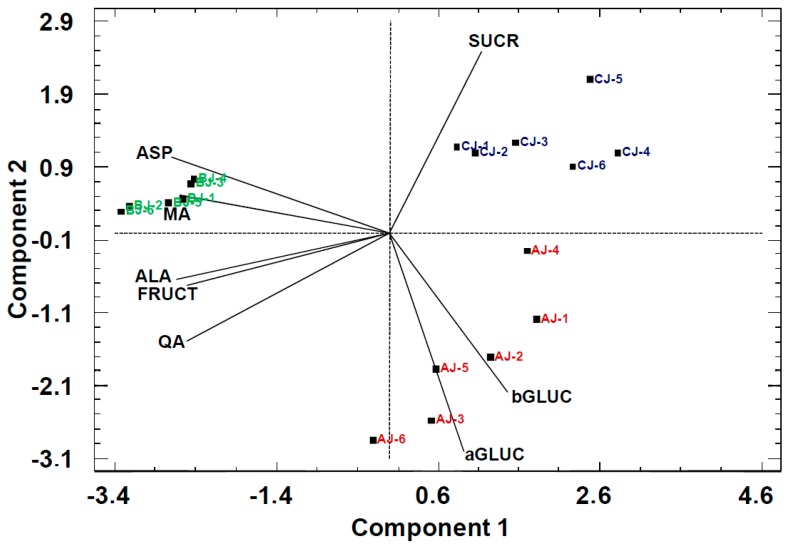
PCA bi-plot of pear juices with primary metabolites as statistical variables. AJ: Alejandrina juice, CJ: Conference juice, and BJ: Blanquilla juice. PC1: 60%, PC2: 24%.

The scores in the PCA bi-plot displayed results largely consistent with the origin of the pear cultivar, discriminating the 18 pear juice batches in three groups, namely Alejandrina (AJ), Blanquilla (BJ), and Conference (CJ) (Figure 2).

The juice scores showed a clear separation in PC1 between Blanquilla (BJ) and Alejandrina (AJ) and Conference (CJ) and, in PC2, between Alejandrina (AJ) and Blanquilla (BJ). The loadings of the variables in the plot show that the separation along PC1 is described by differences in acid content. In this case, the PC1 revealed a strong correlation of organic acids and amino acids (Pearson correlation of 0.70–0.92, *p* < 0.05) and the samples with high acid content belonging to the Blanquilla (BJ) cultivar. In contrast, the separation on PC2 was based on the strong negative correlation between sucrose (SUCR) and glucose (αGLC and βGLC).

### 2.2. Chemometric Analysis of Phenolic Compounds Determined by Ultra Performance Liquid Chromatography coupled to Photodiode Array, Fluorescence and Tandem Mass Spectrometry UPLC-PDA-FLR-MS/MS

Table 2 summarizes the chromatography, ultraviolet–visible spectroscopy (UV-vis), and Mass Spectrometry (MS) parameters of the phenolic compounds in the samples. The identification of these compounds in the samples was carried out on the basis of available standards and other tools that are useful to identify the maximum number of compounds. The chromatographic behavior and UV-vis absorption spectra, together with published data on the main phenolic compounds in the fruit studied, were used for identification purposes. We confirmed the structures of the phenolic compounds detected using the molecular ion and fragmentation pathways of MS.

Caffeoylquinic and *p*-coumaroylquinic acids were identified in the derivatives of both fruits. The isomers 3, 4, and 5 of *p*-coumaroylquinic and caffeoylquinic acids were distinguished on the basis of the product ion spectra from [M − H]^−^ and relative intensities of their ions. The characteristic ions were 191, 173, and 163 *m*/*z* in *p*-coumaroylquinic acid isomers and 191, 179, and 173 *m*/*z* in caffeoylquinic acid isomers [23]. Flavan-3-ols were identified from the standards and confirmed in the samples on the basis of their characteristic fragmentation ions. The fluorescence response allowed a more selective and sensitive integration of compounds (λex = 280; λem = 310 nm). Flavonoids are a widespread family of phenolic compounds, and we had access to at least one standard of each of the main families present in the fruits studied. UV-vis and mass spectra of these standards provided an effective tool for distinguishing the aglycone family and allowed us to identify the flavonoids present. To tentatively identify compounds for which no standards were available, practical guidelines for the characterization of glycosyl flavonoids by triple quadrupole MS were followed [24,25]. In addition, studies reporting the flavonoid profiles of the fruits studied were useful for this purpose [13,15,26,27]. In peach purees, glycoside flavonols with the aglycones quercetin and kaempferol were identified, which have the characteristic fragments 303/301 for quercetin glycosides and 287/285 for kaempferol glycosides in positive and negative mode, respectively. Moreover, flavonol glycosides belonging to isorhamnetin aglycone were detected in pear juices, which presented the fragment 317/315 due to the loss of sugar in positive and negative mode, respectively. 

A mix of standard compounds with one standard of each family (arbutin (Arb), 5-caffeoylquinic acid (5-Caf-qui), catechin (Cat), and quercetin-3-*O*-glucoside (Q-glu)) was injected once every ten injections to control stability during the injection set. The areas of all peaks selected in the chromatograms of samples were normalized against the area of the corresponding standard of the same family of compounds injected in the sample set. The key figures were tabulated and used to build the PCA models. The weight of variables was normalized by the ratio of their standard deviation in the data set. Scores and loadings in the bi-plots obtained in the PCA model were used to determine whether the phenolic compound profiles of each juice sample were sufficient to distinguish between the juice of pear varieties and between the puree of peach varieties, as well as to identify markers for each variety.

**Figure 3:**
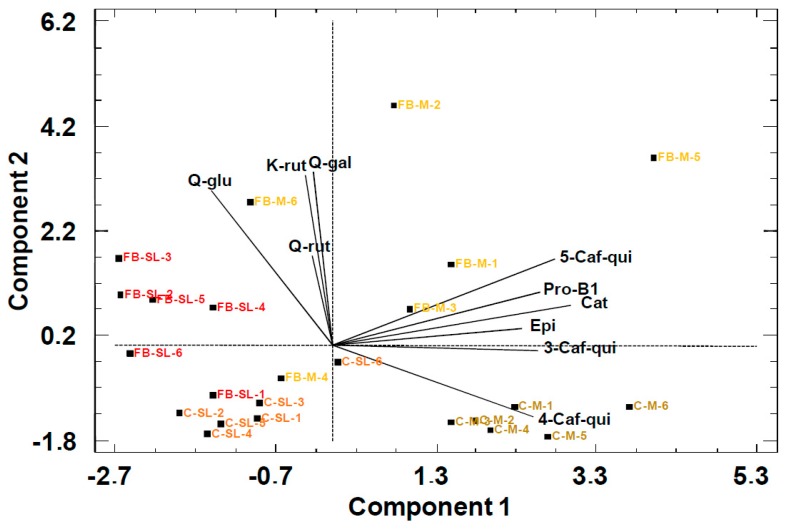
PCA bi-plot of peach purees with phenolic compounds as statistical variables. FB: Freshly blended, C: Commercial, SL: Spring Lady cultivar, and M: Miraflores cultivar. Ending number is the batch number. PC1: 42%, PC2: 32%.

In the model built from the chromatograms of peach purees, the first two PCs (PC1 and PC2) were selected to provide the highest variation of data objects (42% PC1 and 32% PC2). The scores of the PCA bi-plot (Figure 3) produced a moderate separation through PC1 of the commercial (C) and freshly blended (FB) purees of both the Miraflores (M) and Spring Lady (SL) cultivars.

The loadings of the variables in the plot show that the separation was based on flavonol glycosides (Q-gal, Q-gluc, Q-rut, and K-rut) against the other phenolic compounds (Pearson correlation between –0.68 and –0.12, *p* < 0.05). The separation between the cultivars was better in commercial (C) purees than in freshly blended (FB). The sample of freshly blended Miraflores puree from batch four (FB-M-4) was crossed in the PCA model with samples of commercial Spring Lady puree (C-SP). The FB-M-4 sample already showed lower separation in the PCA model by NMR. This observation could be attributed to the fact that sampling is never as thorough in a fruit batch as in a juice batch because the latter includes a homogenization step. In contrast to the former model with primary metabolites, this new model produced separation between commercial (C) and the freshly blended (FB) peach purees. Separation between these two types of commercial (C) and freshly blended (FB) purees was performed along PC2. Freshly blended samples (FB) samples showed a greater concentration of phenolic compounds than commercial samples (C). These results are in accordance with the degradation of polyphenols during the thermal and blending steps of fruit processing [18,21,28].

**Figure 4:**
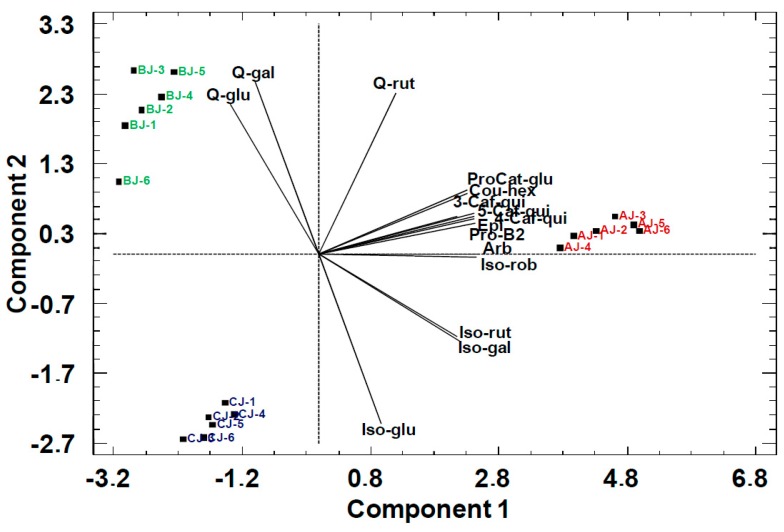
PCA bi-plot of pear juices with phenolic compounds as statistical variables. AJ: Alejandrina juice, CJ: Conference juice, and BJ: Blanquilla juice. PC1: 71%, PC2: 25%.

The scores of the PCA bi-plot for pear juices (Figure 4) showed an improved separation over the model of primary metabolites obtained by ^1^H NMR. 

In the model built from the phenolic compound constituents of pear juices, isorhamnetin 3-O-malonygalactoside and isorhamnetin 3-*O*-6″-malonygalactoside (Iso-6″-malgal) were not included because they are not present in the juice of the C variety. In the model plotted, the first two PCs explained 96% of the information contained in the data (71% PC1 and 25% PC2). The hydroxycinnamic acids (3-Caf-qui, 5-Caf-qui, 4-Caf-qui) were strongly correlated among them and with arbutin (Arb). Further, in PC1 they were correlated with isorhamnetin glycosides (Iso-rut, Iso-gal, Iso-glu). In contrast, quercetin galactoside and glucoside (Q-gal and Q-glu) were anti-correlated with hydroxycinnamic acids (3-Caf-qui, 5-Caf-qui, 4-Caf-qui) through PC1, which allowed the separation of Alejandrina (AJ) from the other two cultivars. The negative correlation between isorharmnetin glycosides (Iso-rut, Iso-gal) and quercetin glycosides (Q-gal and Q-glu) in the PC2 allowed the separation between the Blanquilla (BJ) and Conference (CJ) cultivars. 

### 2.3. Combination of ^1^H NMR and UPLC-PDA-FLR-MS/MS Chemometric Analysis

**Figure 5:**
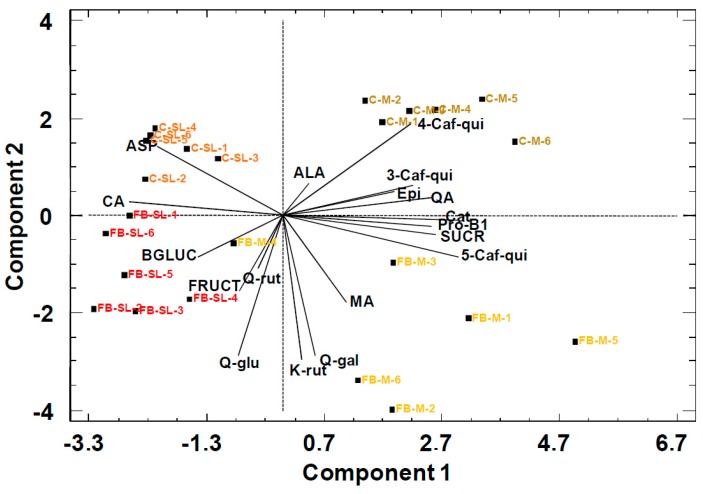
PCA bi-plot of peach purees with primary metabolites and phenolic compounds as statistical variables. FB: Freshly blended, C: Commercial, SL: Spring Lady cultivar, and M: Miraflores cultivar. Ending number is the batch number. PC1: 37%, PC2: 22%.

**Figure 6:**
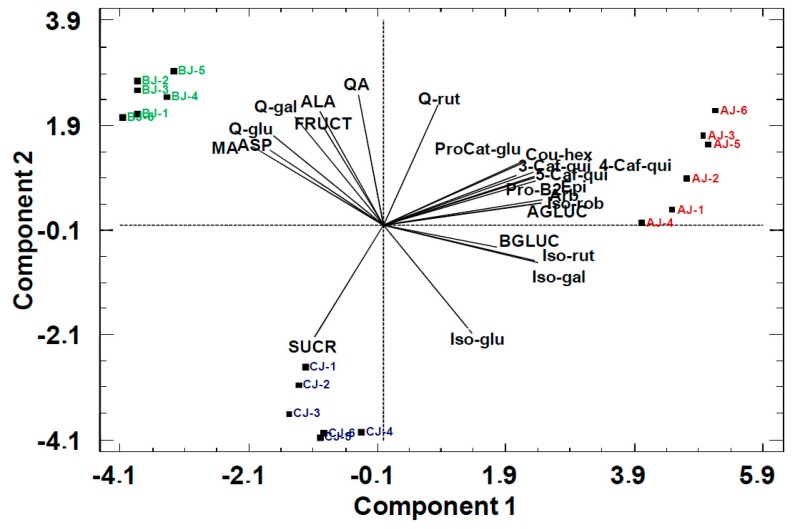
PCA bi-plot of pear juices with primary metabolites and phenolic compounds as statistical variables. AJ: Alejandrina juice, CJ: Conference juice, and BJ: Blanquilla juice. PC1: 58%, PC2: 32%.

The PCA models of peach puree and pear juices (Figure 5 and Figure 6) were built by combining data of primary metabolites and phenolic compounds, in order to evaluate the improvement in separation obtained. In both models, the first two PCs provided the highest variation of data objects (PC1 and PC2, respectively, accounting for 37% and 22% of the information for peach purees, and 58% and 32% for pear juices).

The scores of the PCA bi-plot combination for peach purees (Figure 5) substantially improved the separation with respect to the separate models for primary metabolites and phenolic compounds. The separation was achieved through PC1 component between the Miraflores (M) and Spring Lady (SL) cultivars both commercial (C) and freshly blended (FB) purees. Only the outlier FB-M-4 continued to be separated from the other samples of freshly blended puree from Miraflores cultivar. 

The loadings of the variables in this plot showed that the separation was based on asparagine (ASP) and citric acid (CA) against sucrose (SUCR), quinic acid(QA), epicatechin (Epi), catechin (Cat) and various hydroxycinnamics acids (Pearson correlation between –0.68 and –0.12, *p* < 0.05). The inclusion of the phenolic compounds in the primary metabolite model allowed the separation of commercial (C) and freshly blended (FB) purees.

Regarding the pear juices (Figure 6), the PCA bi-plot combination improved the density of scores of juice from the Blanquilla cultivar, but did not affect the separation obtained in the previous models. Indeed, the three cultivars were separated along the PC1.

## 3. Materials and Methods

### 3.1. Chemicals

Methanol (MeOH) and acetone (HPLC grade purity) were supplied by J.T. Baker (Deventer, The Netherlands). The chromatographic solvents were acetonitrile (ACN) (LC-MS grade), also from J.T. Baker, and water, purified in a Milli-Q system from Millipore (Bedford, MA, USA). The chromatographic eluent additive was acetic acid (HAcO) (LC-MS grade), provided by Fluka (Sigma-Aldrich, Madrid, Spain), and formic acid (HFor) and hydrochloric acid 37% (analytical grade) were supplied by Merck. Sodium hydroxide was from Panreac (Barcelona, Spain) and ascorbic acid from Acros (Pittsburgh, PA, USA). Citric acid, malic acid, quinic acid, fructose, glucose, sucrose, and amino acids were supplied by Fluka. 

The phenolic standards (+)-catechin, (−)-epicatechin, procyanidin B1, procyanidin B2, arbutin, eriodictyol-7-*O*-rutinoside, naringenin-7-*O*-rutinoside, naringenin, hesperetin-7-*O*-rutinoside, quercetin-3-*O*-galactoside, quercetin-3-*O*-glucoside, quercetin-3-*O*-rhamnoside, quercetin-3-*O*-rutinoside, kaempferol-3-*O*-rutinoside, and isorhamnetin-3-*O*-rutinoside were supplied by Extrasynthèse (Genay, France). Arbutin, gallic acid, phloretin-2′-*O*-β-glucoside, 5′-caffeoylquinic acid, caffeic acid, *p*-coumaric acid, ferulic acid, and sinapic acid were purchased from Sigma–Aldrich Chemie (Steinheim, Germany). All stock standard solutions of phenolic compounds were prepared in methanol and stored at –80 °C. Working solutions were prepared from stock solutions by sampling an aliquot and diluting it with the injection solvent (0.1% acetic acid aqueous solution).

### 3.2. Samples

Six commercial peach purees (Prunus persica) of Spring Lady and Miraflores cultivars and their corresponding fresh fruit were provided by a local fruit juice company (Zucasa, Fraga, Spain). Peaches were stoned and four pieces were homogenized in a blender (Grindomix GM 200; Retsch, Haan, Germany) at 3300× *g* for 2 min. Ascorbic acid (approximately 10 g/kg) was added to prevent oxidation in this blending step. The same company provided six pear juices derived from Alejandrina, Conference, and Blanquilla cultivars.

The following codes were assigned to the peach puree samples: FB: Freshly blended; C: Commercial; SL: Spring Lady cultivar; and M: Miraflores cultivar. An ending number was added to indicate the batch number (from 1 to 6 peach purees).

The following codes were assigned to the pear juice samples from the distinct cultivars: AJ: Alejandrina juice; CJ: Conference juice; and BJ: Blanquilla juice. An ending number was added to indicate the batch number (from 1 to 6 pear juices).

### 3.3. Sample Preparation

Samples for the NMR analysis: 45 g of juice or puree was clarified by centrifugation (13,500× *g*, 30 min, Hettich EBA 21(Tuttlingen, Germany). Maleic acid was added as internal standard at a concentration of 15 g/L. The supernatant was adjusted to pH 6.5 by addition of NaOH (5 M, 1 M, and 0.1 M). The samples were filtered using a 0.22 µm Polytetrafluoroethylene (PTFE) filter prior to recording the spectrum.

Samples for the UPLC analysis: 2 g of juice or puree was centrifuged at 30,400× *g* (Hettich Eppendorf Centrifuge MIKRO 22 R; Germany). Then 0.5 mL of the supernatant was diluted 1:2 with Milli-Q water (acidified at 0.1% *v*/*v* with acetic acid) and filtered using a 0.22 µm PTFE filter. Solutions were kept at 6 °C until UPLC analysis. All solvents contained ascorbic acid (0.2% *w*/*v*).

### 3.4. H NMR Analysis

A volume of 600 μL of the sample (from juice or puree samples) and 100 µL of D_2_O were introduced into an NMR tube with an outer diameter of 5 mm. One-dimensional spectra were recorded on a Varian AS 400 spectrometer operating at 400 MHz. The following acquisition parameters were used: spectral width 6402 Hz; relaxation delay 15 s; number of scans 128; acquisition time 4.09 s; and pulse width 90°. Experiments were carried out at 25 °C. The recycle delay between scans was set to 5T_1_ of the malic acid, the longest value of T_1_. T_1_ relaxation time was determined using the inversion recovery pulse sequence. This procedure ensured complete relaxation of the molecules present in the fruit juices and of the internal standard from the NMR signals. Spectra were Fourier-transformed and weighted with a Gaussian function. Baseline correction was applied across the whole spectral range.

### 3.5. UPLC Analysis. UPLC-PDA-FLR Parameters

Ultra-performance liquid chromatographic analysis was performed on a Waters ACQUITY UPLC™ system (Waters, Milford, MA, USA) consisting of an ACQUITY UPLC™ binary solvent manager and ACQUITY UPLC™ sample manager, coupled to a photodiode array detector ACQUITY UPLC™ PDA and fluorescence detector ACQUITY UPLC™ FLR. Compounds were separated with an ACQUITY UPLC™ HSS T3 column (1.8 µm; 2.1 mm × 150 mm) (Waters, Manchester, UK) using a mobile phase consisting of solvent A, H_2_O (0.1% *v*/*v* HAcO), and solvent B, ACN 100% (0.1% *v*/*v* HAcO). The flow rate was 0.55 mL/min. The linear gradient was as follows: 0–1.89 min, 1% B, (isocratic); 1.89–17.84 min, 30% B, (linear gradient); 17.84–21.39 min, 5% B, (linear gradient); 21.39–21.56 min, 1% B, (linear gradient); and 21.56–25 min, 1% B, (isocratic). Weak and strong needle solvents were H_2_O (0.1% *v*/*v* HAcO) and MeOH, respectively. The injection volume was 20 µL in full loop mode, and the temperature in the column was kept at 45 °C and that in the sample injector at 10 °C.

Tables were built by noting chromatographic peak areas of the following phenolic families measured at wavelengths: 320 nm for hydroxycinnamic acids; 283 nm for flavanones; 285 nm for hydroquinone; and 340 nm and 350 nm for flavonols (isorhamnetin, kaempferol, and quercetin glycosides). Fluorescence values for flavan-3-ols were measured at 310 nm (λex = 280 nm). UPLC chromatograms are included in the Supplementary Materials.

### 3.6. UPLC-MS/MS Parameters

MS analysis was carried out on a Waters ACQUITY TQD tandem quadrupole mass spectrometer (Waters, UK). The instrument was operated using an electrospray source (ESI) in positive and negative ion mode. The following ESI parameters were used: capillary voltages of 3.5 kV and –2.5 kV in positive and negative mode, respectively; the source at 150 °C; desolvation temperature at 500 °C; cone gas (nitrogen) flow of 50 L/h; and desolvation gas flow of 800 L/h. Flow injections of each individual standard were used to optimize the cone voltage and multiple reaction monitoring (MRM) parameters. Collision-induced dissociation was achieved using argon at a flow rate of 0.15 mL/min in the collision cell. MS conditions of standard compounds with same aglycone were applied to identify compounds for which standards are not available. MassLynx 4.1 software (Waters, Milford, MA, USA) was used for data acquisition.

### 3.7. Chemometric Analysis

Chemometric analysis was performed using Statgraphics Centurion XVI Version: 16.2.04 (StatPoint Technologies, Warrenton, VA, USA). Correlation analysis was carried out to determine the relationships between the main components. We used Principal Component Analysis (PCA) as the projection technique for the multivariate analysis of data. Analysis of Variance ANOVA test results and Venn diagrams are included in the Supplementary Materials.

## 4. Conclusions

The present study shows an approach for the authentication of monovarietal peach purees and pear juices on the basis of primary metabolites and phenolic compounds. The PCA of primary metabolites, from ^1^H-NMR data, allowed the discrimination between of different varieties in the case of pear and peach, but did not allow to discriminate between processed and non-processed purees. On the other hand, PCA approaches of phenolic compounds analyzed by UPLC-PDA-MS/MS discriminated between varieties in peach purees and pear juices and also allowed to discriminate between processed and non-processed peach purees. PCA combined data of primary metabolites and phenolic compounds improved the separation of clusters in all cases.

## Figures and Tables

**Table 1:** Summary of the ^1^H NMR signals for the spectra of pear and peach used for the statistical analyses.

Compound ^2^	Assignment	^1^H (ppm Hz)
Alanine (ALA)	β-CH_3_	1.49
Quinic acid (QA)	CH_2_-1,1′; CH_2_-5,5′	1.7
Citric acid (CA)	α,γ-CH	2.3
Asparagine (ASP)	β, β′-CH_2_	2.7
β-Glucose (βGLC)	CH-2	2.95
β-D-Fructopyranose (FRU)	CH_2_-6,6′	3.85
Malic acid (MA)	β, β′-CH_2_	4.1
α-Glucose (αGLC)	CH-1	4.95
Sucrose (SUCR)	GLC CH-1	5.15
Maleic acid (IS)	2CH	5.9

^2^ The abbreviation for each compound is shown in brackets.

**Table 2:** The chromatographic, UV-vis, and MS parameters of the phenolic compounds identified in the samples.

Retention Time (min)	Compound^1^	UV Max (nm)	[M − H]^−^/[M + H]^+^ (*m*/*z*)	Relative Abundances of Main Ions from MS/MS of [M − H]^−^/[M + H]^+^	CE (eV)^4^
2.53	Hydroquinone β-d-glucopyranoside or Arbutin^2^ (Arb)	283	271/273	108(100), 95 (18)/−	20
5.85	Protocatechuic aldehyde monoglucuronide^3^ (ProCat-glu)	268	313/315	175/−	20
6.45	3-Caffeoylquinic acid^3^ (3-Caf-qui)	240, 326	353/355	191 (100), 179 (48)/−/	20
7.02	Coumaroyl-hexose^3^ (Cou-hex)	316	325/327	163/-	20
7.68	Procyanidin-B1^2^ (Pro-B1)	278	289/291	407(100), 289(63), 425 (34),/291(100), 409(88), 427(64), 247(64), 301(64)	20
7.96	5-Caffeoylquinic acid^2^ (5-Caf-qui)	240, 326	353/356	191 (100), 179 (94)/163(100), 145(62), 135(20), 117(20)	20
8.22	(+)-Catechin^2^ (Cat)	278	289/291	139(100), 123(47), 147(22)/203(100), 109(94), 125(84)	20
8.43	4-Caffeoylquinic acid^3^ (4-Caf-qui)	240, 326	353/355	173 (100), 179(67),191(32) /−	20
9.23	Procyanidin-B2^2^ (Pro-B2)	278	577/579	291(100), 409(88), 427(64), 247(64), 301(64) /407(100), 289(63), 425 (34)	20
9.47	5-*p*-Coumaroylquinic acid^3^ (5-*p*-Cou-qui)	235,312	337/339	191(100), 163(22)/−	20
9.88	(-)-Epicatechin^2^ (Epi)	278	289/291	139(100), 123(47), 147(22)/203(100), 109(94), 125(84)	20
12.49	Quercetin-3-*O*-rutinoside^2^ (Q-rut)	255, 353	609/611	300(100)/303(100)	30
12.59	Quercetin-3-*O*-galactoside^2^ (Q-gal)	255, 353	463/465	300(100), 271(27), 255(19)/303(100), 153(26)	30
12.82	Quercetin-3-*O*-glucoside^2^ (Q-glu)	255, 353	463/465	300(100), 271(27), 255(19)/303(100), 153(26)	30
13.64	Kaempferol 3-*O*-rutinoside^2^ (K-rut)	265, 347	593/595	287/−	30
13.8	Isorhamnetin-3-*O*-robinioside^3^ (Iso-rob)	254, 352	623/625	317/−	30
13.98	Isorhamnetin-3-*O*-rutinoside^2^ (Iso-rut)	254, 352	623/625	317/−	30
14.12	Isorhamnetin-3-*O*-galactoside^3^ (Iso-gal)	254, 352	477/479	317/−	30
14.38	Isorhamnetin-3-*O*-glucoside^3^ (Iso-glu)	254, 352	477/479	317/−	30
15.05	Isorhamnetin 3-*O*-malonygalactoside^3^ (Iso-malgal)	254, 352	563/565	317/−	30
15.36	Isorhamnetin 3-*O*-6″-malonygalactoside^3^ (Iso-6″-malgal)	254, 352	563/565	317/−	30

^1^ The abbreviation used for each compound is indicate in brackets. ^2^ Identification on the basis of the UV-vis spectrum and mass fragments specific to the standard compounds. ^3^ Tentative identification based on UV-vis spectrum, MS, HPLC/UHPLC retention times and published data. ^4^ Collision _Energy.

## Supplementary Materials

The following are available online. Phenolic compounds chromatograms page 2, ^1^H NMR spectra page 4, ANOVA results page 8, Venn diagrams page 10.

## Abbreviations

FB: Freshly blended; C: Commercial; SL: Spring Lady cultivar; M: Miraflores cultivar; AJ: Alejandrina juice; CJ: Conference juice; BJ: Blanquilla juice; IS: internal standard; ALA: alanine; QA: quinic acid; CA: citric acid; ASP: asparagine; βGLC: β-glucose; FRU: β-d-fructopyranose; MA: malic acid; αGLC: α-glucose; SUCR: sucrose; Arb: Hydroquinone β-d-glucopyranoside or Arbutin; ProCat-glu: Protocatechin aldehyde monoglucuronide; 3-Caf-qui: 3-Caffeoylquinic acid; Cou-hex: Coumaroyl-hexose; Pro-B1: Procyanidin-B1; 5-Caf-qui: 5-Caffeoylquinic acid; Cat: (+)-Catechin; 4-Caf-qui: 4-Caffeoylquinic acid; Pro-B2: Procyanidin-B2; 5-*p*-Cou-qui: 5-*p*-Coumaroylquinic acid; Epi: (-)-Epicatechin; Q-rut: Quercetin-3-*O*-rutinoside; Q-gal: Quercetin-3-*O*-galactoside; Q-glu: Quercetin-3-*O*-glucoside; K-rut: Kaempferol 3-*O*-rutinoside; Iso-rob: Isorhamnetin-3-*O*-robinioside; Iso-rut: Isorhamnetin-3-*O*-rutinoside; Iso-gal: Isorhamnetin-3-*O*-galactoside; Iso-glu: Isorhamnetin-3-*O*-glucoside; Iso-malgal: Isorhamnetin 3-*O*-malonygalactoside; Iso-6″-malgal: Isorhamnetin 3-O-6″-malonygalactoside.
