# Supplementary material for: Combined Analysis of Primary Metabolites and Phenolic Compounds to Authenticate Commercial Monovarietal Peach Purees and Pear Juices
Source: Molecules. 2019 Sep 10;24(18):3289. doi: 10.3390/molecules24183289 (PMC6767303; doi:10.3390/molecules24183289)
Supplement: Supplementary file 1 [file molecules-24-03289-s001.pdf]

SUPPLEMENTARY MATERIAL

INDEX

PHENOLIC COMPOUNDS CHROMATOGRAMS	S2
ION SPECTRA FROM CAFFEOYLQUINIC ACID ISOMERS	S5
^{1}H NMR SPECTRA	S6
ANOVA RESULTS	S10
VENN DIAGRAMS	S12

PHENOLIC COMPOUNDS CHROMATOGRAMS

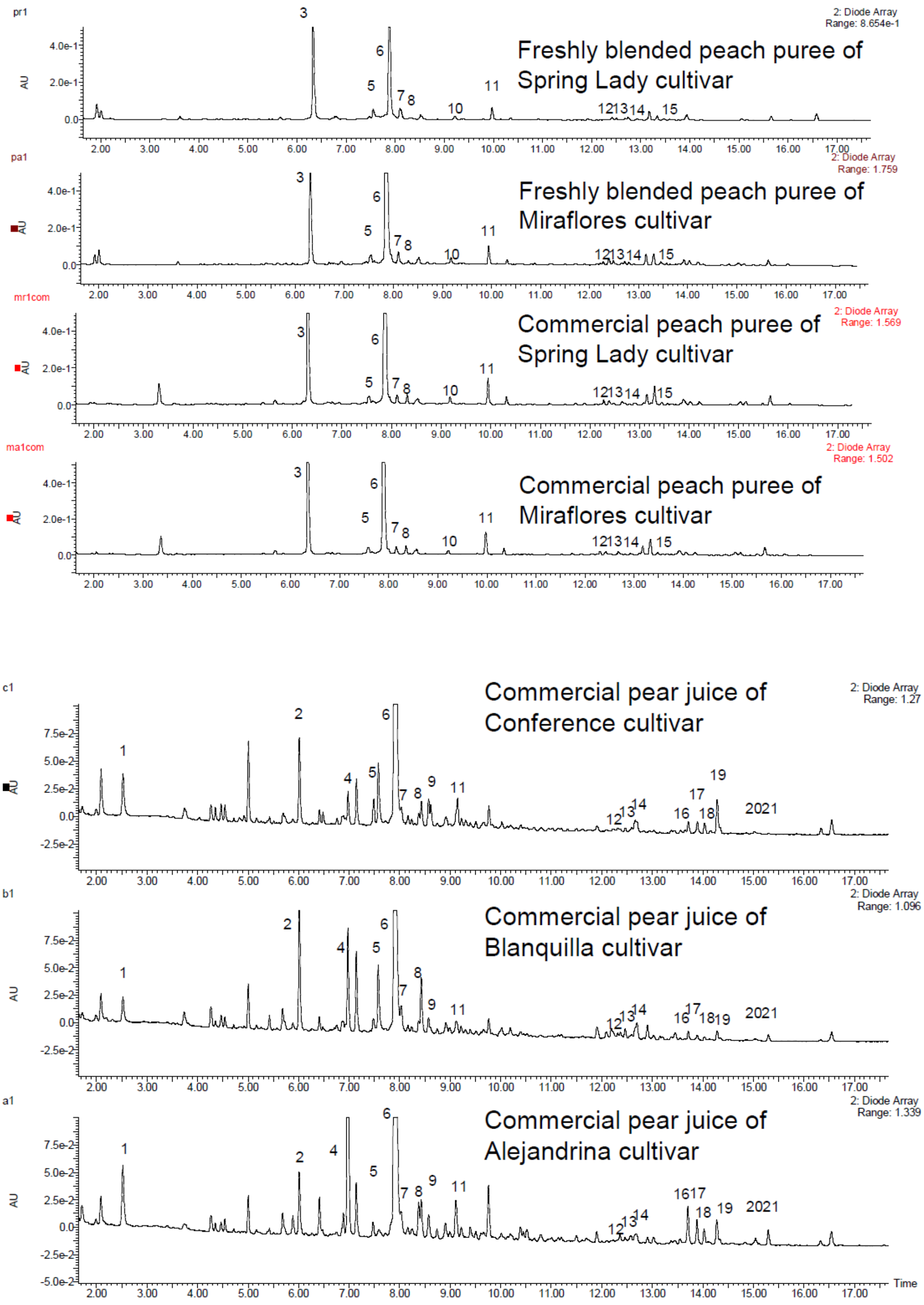

Figure 1. Chromatograms at 350 + 280 + 320 nm of pear juices and peach purees. Numbered according the next table.

Peak number	Compound	UV max (nm)
1	Hydroquinone β-D-glucopyranoside or Arbutin [a] (Arb)	283
2	Protocatechuic aldehyde monoglucuronide [b] (ProCat-glu)	268
3	3-Caffeoylquinic acid [b] (3-Caf-qui)	240, 326
4	Coumaroyl-hexose [b] (Cou-hex)	316
5	Procyanidin-B1 [a] (Pro-B1)	278
6	5-Caffeoylquinic acid [a] (5-Caf-qui)	240, 326
7	(+)-Catechin [a] (Cat)	278
8	4-Caffeoylquinic acid [b] (4-Caf-qui)	240, 326
9	Procyanidin-B2 [a] (Pro-B2)	278
10	5-*p*-Coumaroylquinic acid [b] (5-p-Cou-qui)	235, 312
11	(-)-Epicatechin [a] (Epi)	278
12	Quercetin-3-*O*-rutinoside [a] (Q-rut)	255, 353
13	Quercetin-3-*O*-galactoside [a] (Q-gal)	255, 353
14	Quercetin-3-*O*-glucoside [a] (Q-glu)	255, 353
15	Kaempferol 3-*O*-rutinoside [a] (K-rut)	265, 347
16	Isorhamnetin-3-*O*-robinioside [b] (Iso-rob)	254, 352
17	Isorhamnetin-3-*O*-rutinoside [a] (Iso-rut)	254, 352
18	Isorhamnetin-3-*O*-galactoside [b] (Iso-gal)	254, 352
19	Isorhamnetin-3-*O*-glucoside [b] (Iso-glu)	254, 352
20	Isorhamnetin 3-*O*-malonygalactoside [b] (Iso-malgal)	254, 352
21	Isorhamnetin 3-*O*-6′′-malonygalactoside [b] (Iso-6′′-malgal)	254, 352

ION SPECTRA FROM CAFFEOYLQUINIC ACID ISOMERS

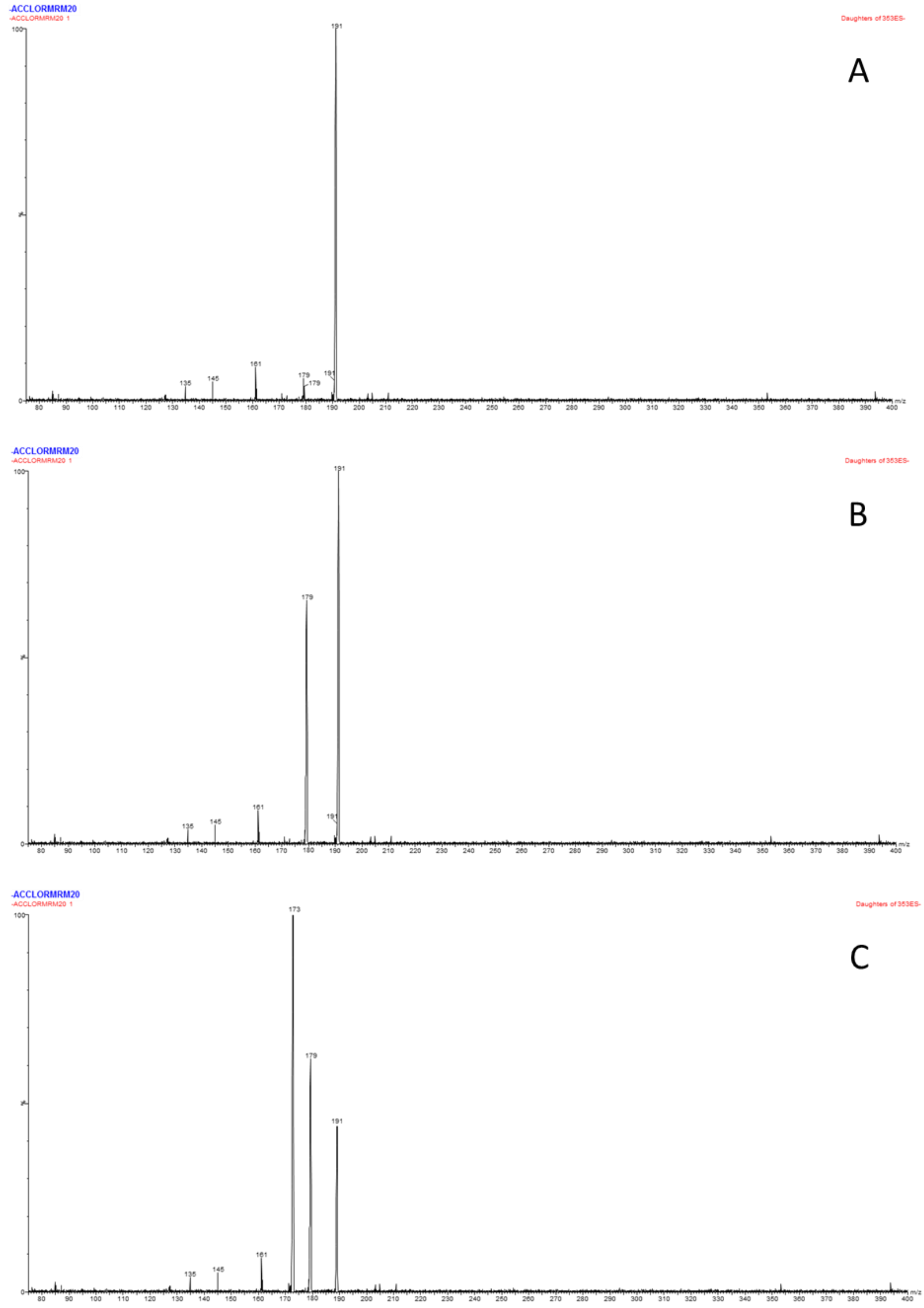

Figure 2. Ion spectra of caffeoylquinic isomers: **A**: 5-Caffeoylquinic acid (5-Caf-qui), **B**: 3-Caffeoylquinic acid (3-Caf-qui) and **C**: 4-Caffeoylquinic acid (4-Caf-qui).

^{1}H NMR SPECTRA

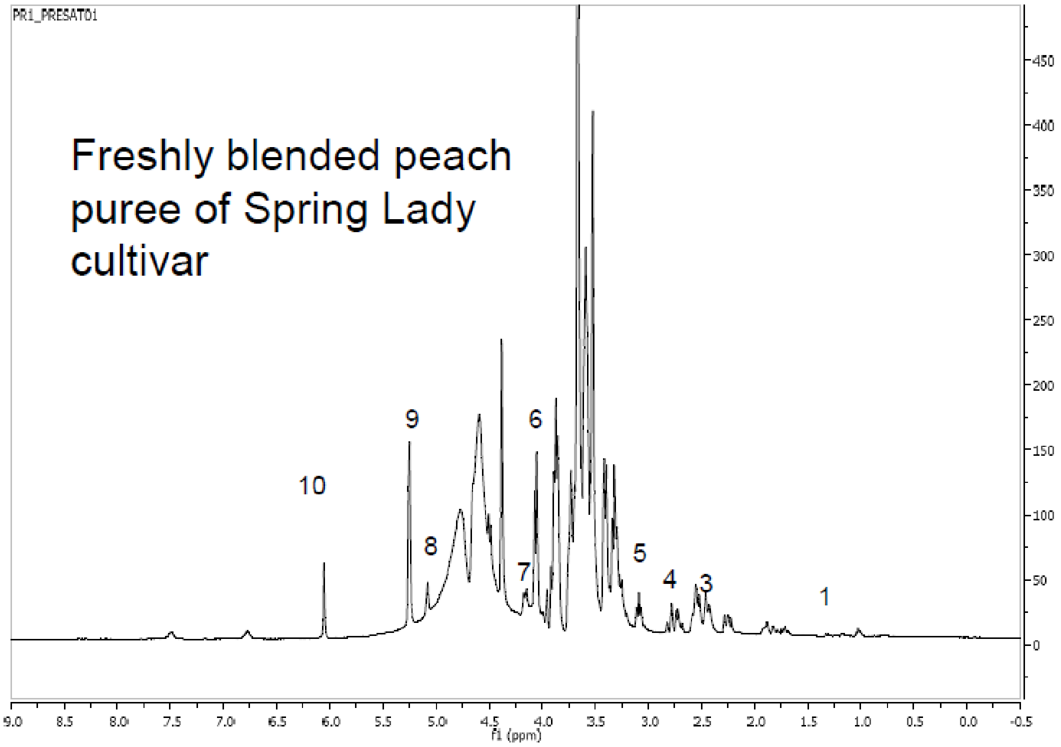

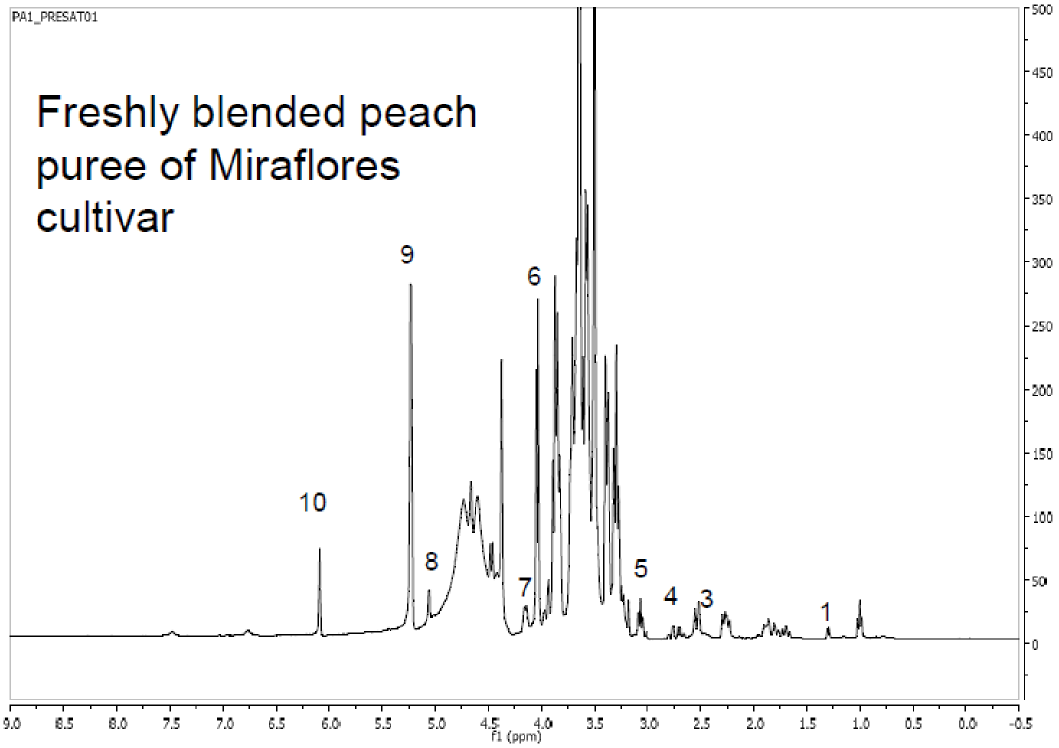

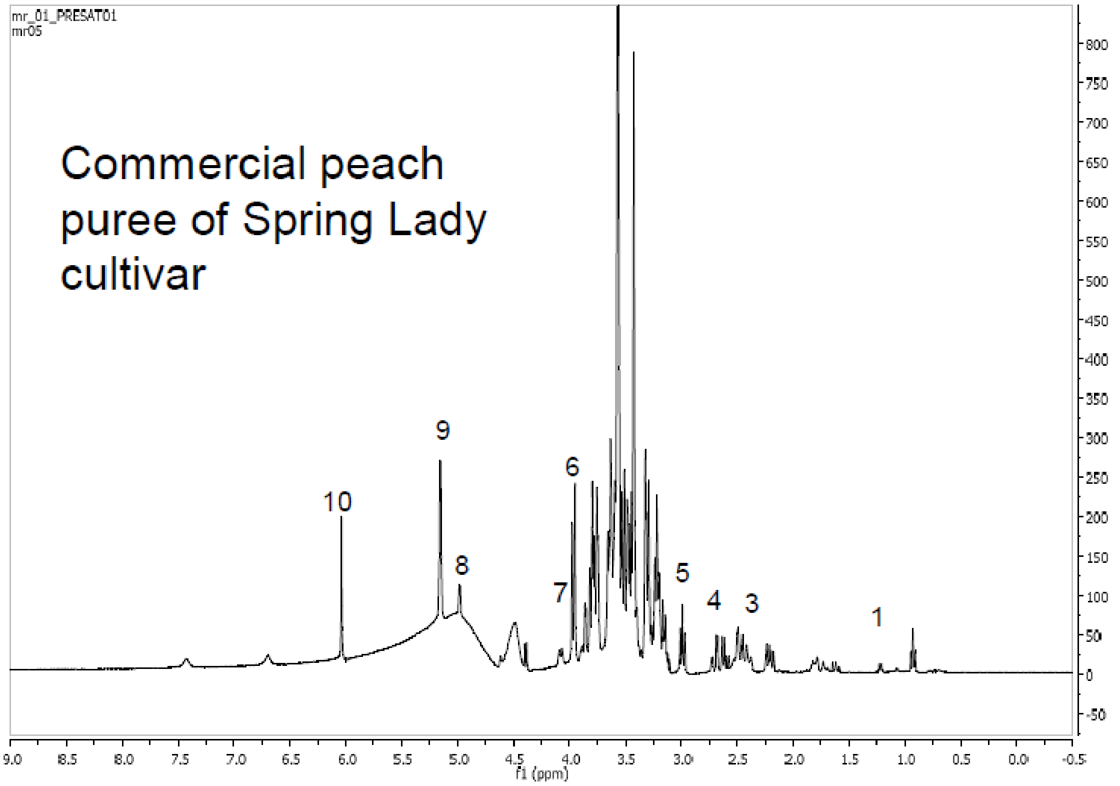
mr_01_PRESAT01
mr05
Commercial peach puree of Spring Lady cultivar
10
9
8
7
6
5
4
3
1
f1 (ppm)

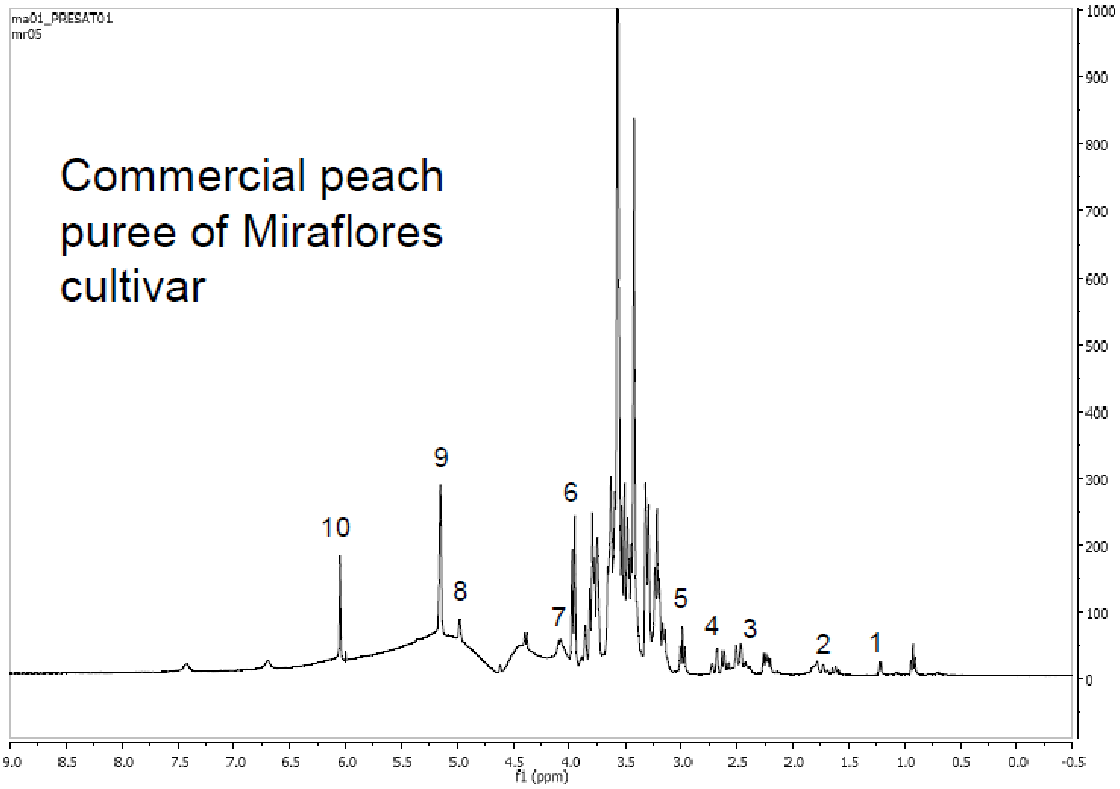
ma01_PRESAT01
mr05
Commercial peach puree of Miraflores cultivar
10
9
8
7
6
5
4
3
2
1
f1 (ppm)

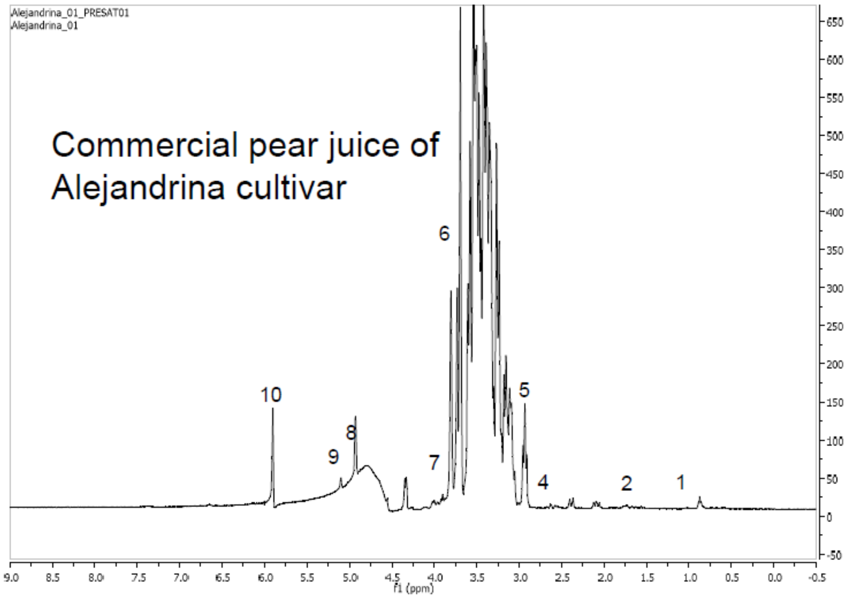

Alejandrina_01_PRESAT01
Alejandrina_01
Commercial pear juice of
Alejandrina cultivar
1
2
4
5
6
7
8
9
10
f1 (ppm)

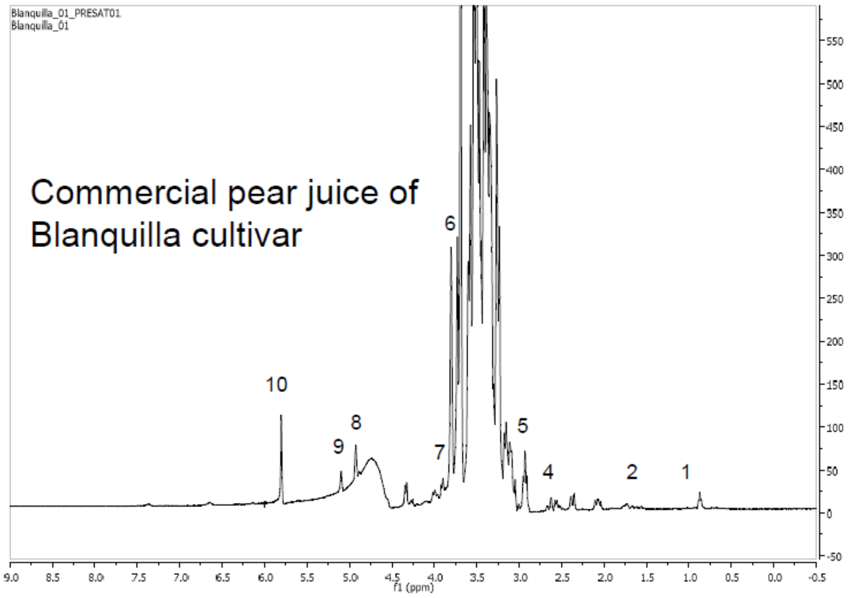

Blanquilla_01_PRESAT01
Blanquilla_01
Commercial pear juice of
Blanquilla cultivar
1
2
4
5
6
7
8
9
10
f1 (ppm)

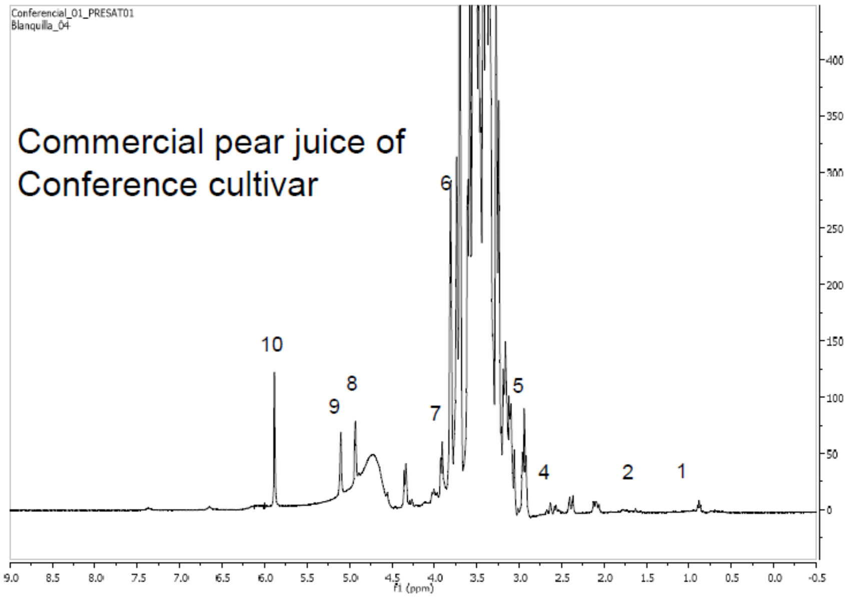

Conferencial_01_PRESAT01
Blanquilla_04
Commercial pear juice of
Conference cultivar
1
2
4
5
6
7
8
9
10
f1 (ppm)

Figure 3. ^{1}H NMR spectra of commercial pear juices and peach purees. Numbered according the next table.

**Signal number**	**Compound**	**Assignment**	**^{1}H (ppm)**
1	Alanine (ALA)	β-CH_3	1.49
2	Quinic acid (QA)	CH_2-1,1′; CH_2-5,5′	1.7
3	Citric acid (CA)	α,γ-CH	2.3
4	Asparagine (ASP)	β, β′-CH_2	2.7
5	β-Glucose (βGLC)	CH-2	2.95
6	β-D-Fructopyranose(FRU)	CH_2-6,6′	3.85
7	Malic acid (MA)	β, β′-CH_2	4.1
8	α-Glucose (αGLC)	CH-1	4.95
9	Sucrose (SUCR)	GLC CH-1	5.15
10	Maleic acid (IS)	2CH	5.9

ANOVA RESULTS

Peach puree samples

Oneway Anova p-value cut-off:.0.05

Compound	p	FC ([PM] vs [PSL])	FC ([PM] vs [UnPM])	FC ([PM] vs [UnPSL])	FC ([PSL] vs [UnPM])	FC ([PSL] vs [UnPSL])	FC ([UnPM] vs [UnPSL])
**SUCR**	1.42E−04	1.48	−1.02	1.43	−1.51	−1.03	1.46
**FRU**	4.94E−02	−1.12	−1.24	−1.22	−1.11	−1.08	1.02
**bGLUC**	2.06E−02	−1.08	−1.05	−1.25	1.03	−1.15	−1.19
**ASP**	3.42E−05	−1.42	1.45	−1.22	2.05	1.16	−1.77
**CA**	1.06E−06	−1.95	1.14	−2.27	2.22	−1.17	−2.59
**MA**	4.35E−03	1.15	−1.23	−1.11	−1.42	−1.28	1.11
**QA**	1.46E−02	1.33	1.14	1.45	−1.17	1.09	1.27
**3-Caf-qui**	1.15E−02	1.46	1.23	1.29	−1.18	−1.13	1.05
**5-Caf-qui**	1.27E−08	1.89	−1.17	2.06	−2.21	1.09	2.41
**4-Caf-qui**	1.10E−08	2.99	2.74	5.85	−1.09	1.96	2.13
**Pro-B1**	1.71E−09	4.43	−1.08	10.13	−4.79	2.28	10.94
**Cat**	2.03E−07	2.47	−1.04	4.55	−2.56	1.84	4.72
**Epi**	1.29E−03	1.49	1.10	3.21	−1.36	2.15	2.93
**Q-gal**	7.63E−06	−1.39	−4.44	−2.61	−3.19	−1.87	1.70
**Q-glu**	9.43E−07	−2.04	−3.80	−4.60	−1.87	−2.26	−1.21
**K-rut**	7.12E−06	1.07	−2.66	−2.45	−2.86	−2.63	1.09

Pear juices samples

Oneway Anova p-value cut-off:.0.05

Compound	p	FC ([Alej] vs [Blanq])	FC ([Alej] vs [Conf])	FC ([Blanq] vs [Conf])
**SUCR**	1.68E−10	−1.83	−4.44	−2.43
**aGLUC**	4.09E−05	1.75	1.49	−1.17
**bGLUC**	4.54E−03	1.62	1.22	−1.33
**FRUCT**	7.30E−03	−1.24	1.10	1.37
**ASP**	4.36E−08	−3.38	−1.30	2.61
**MA**	4.33E−04	−1.55	−1.07	1.44
**QA**	3.93E−08	−1.33	2.16	2.89
**ALA**	5.18E−06	−1.67	1.55	2.58
**Arb**	3.25E−10	2.28	1.86	−1.22
**ProCat-glu**	1.64E−15	2.70	5.22	1.93
**3-Caf-qui**	4.02E−13	3.01	3.81	1.26
**Cou-hex**	6.40E−20	3.56	11.44	3.21
**5-Caf-qui**	2.88E−04	1.39	1.44	1.03
**4-Caf-qui**	1.32E−13	2.92	3.30	1.13
**Pro-B2**	3.21E−10	2.44	2.52	1.03
**Epi**	8.23E−10	2.40	2.63	1.10
**Q-rut**	3.35E−10	1.22	9.08	7.46
**Q-gal**	9.36E−11	−1.97	1.39	2.74
**Q-glu**	1.12E−07	−1.76	−1.00	1.75
**Iso-rob**	4.26E−12	5.24	3.23	−1.62
**Iso-rut**	4.66E−11	6.33	1.60	−3.96
**Iso-gal**	7.60E−15	5.81	1.52	−3.83
**Iso-glu**	3.14E−13	3.73	−1.27	−4.75

Venn Diagram Report

Experiment : NMR-Peach
Active entity list : All Entities

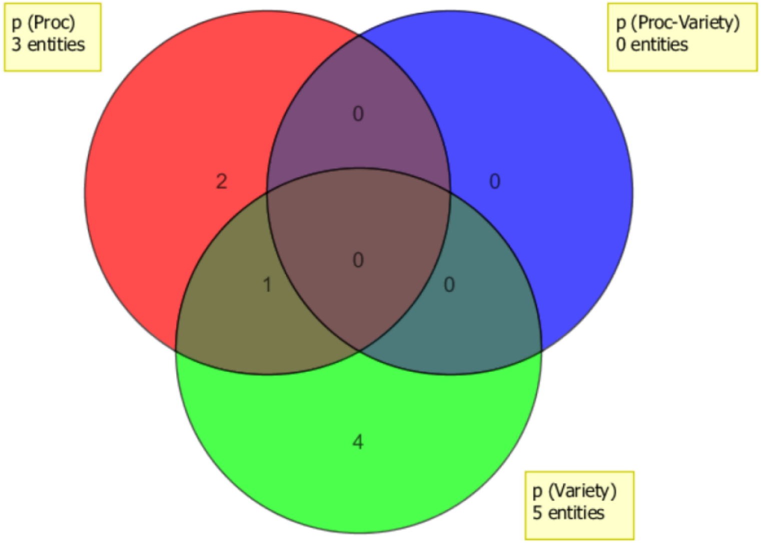

Description
VennDiagram for sets:
p (Proc)
p (Proc-Variety)
p (Variety)

Venn Colors

- p (Proc)
- p (Proc-Variety)
- p (Variety)

Venn Diagram Report

Experiment : Poly-Peach
Active entity list : Oneway ANOVA p cut-off = 0.05

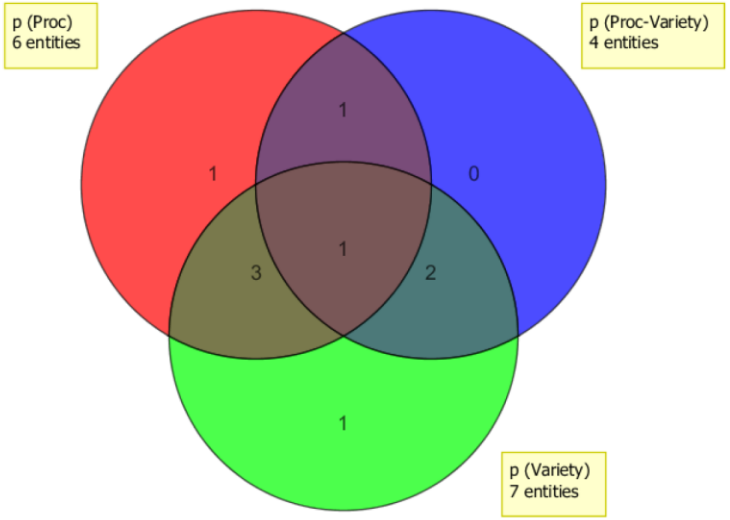

Description
VennDiagram for sets:
p (Proc)
p (Proc-Variety)
p (Variety)

Venn Colors

- p (Proc)
- p (Proc-Variety)
- p (Variety)
